# Supplementary material for: Synergistic and antagonistic activities of IRF8 and FOS enhancer pairs during an immune-cell fate switch
Source: EMBO J. 2025 Feb 19;44(7):2025–55. doi: 10.1038/s44318-025-00380-w (PMC11961672; doi:10.1038/s44318-025-00380-w)
Supplement: Supplementary file 1 — Appendix [file 44318_2025_380_MOESM1_ESM.pdf]

Appendix for

Synergistic and antagonistic activities of IRF8 and FOS enhancer pairs during an immune-cell fate switch

Table of contents

Appendix Figure S1 (Page 1)
Appendix Figure S2 (Page 2)
Appendix Figure S3 (Page 3)
Appendix Figure S4 (Page 4)
Appendix Figure S5 (Page 5)
Appendix Figure S6 (Page 6)
Appendix Figure S7 (Page 7)
Appendix Figure S8 (Page 8)

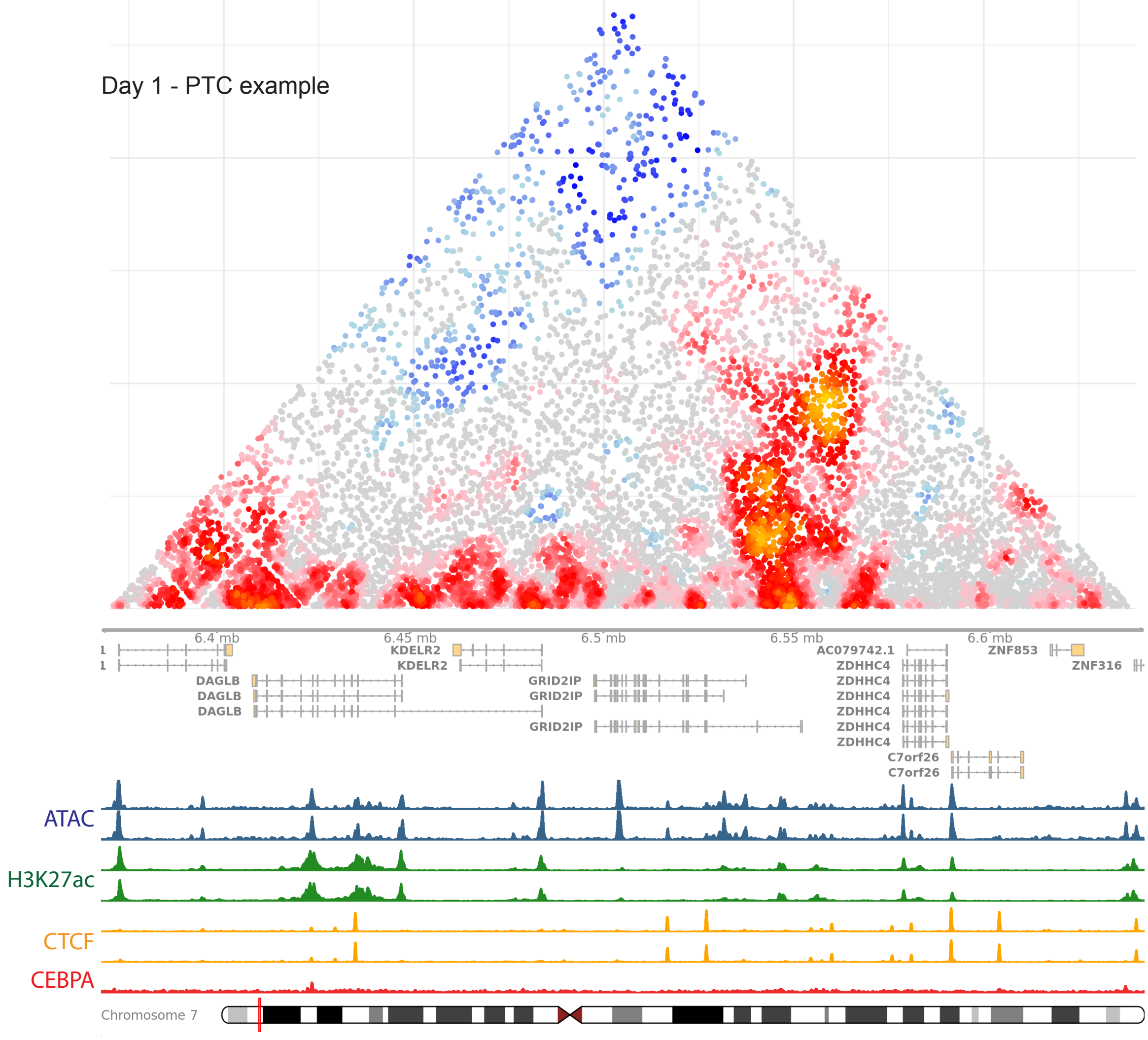

Appendix Figure S1. A Day 1 PTC example region in BLAER cells. SHAMAN (Mendelson Cohen et al., 2017) Hi-C normalized values are depicted, with contact enrichment between loci labeled by red and yellow points and contact depletion by blue points. ATAC-seq and H3K27ac, CTCF, and CEBPA ChIP-seq tracks of two biological replicates are also illustrated. Chromosomal coordinates and genes found within the PTC are also displayed.

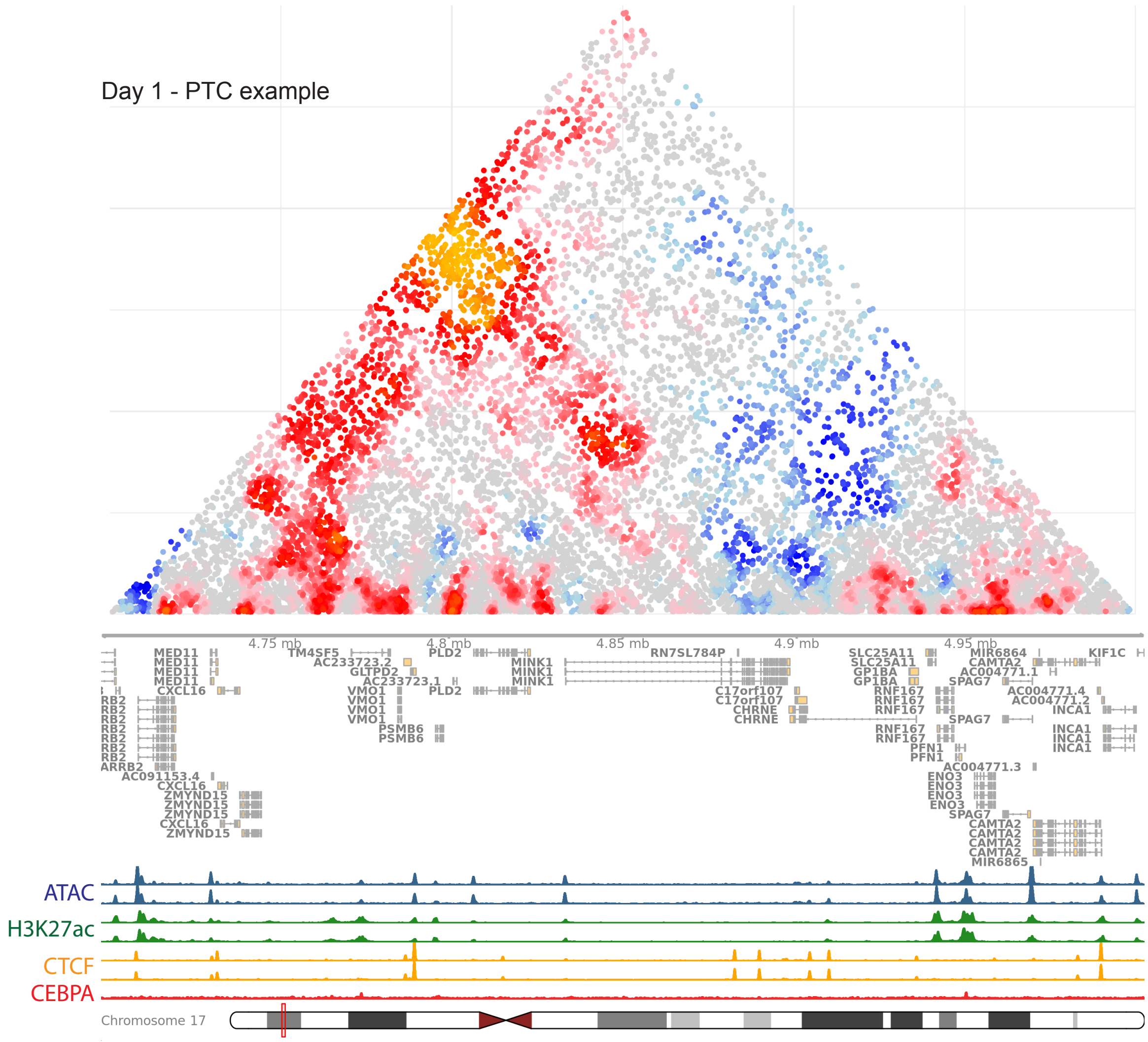

Appendix Figure S2. A Day 1 PTC example region in BLAER cells. Same as in Appendix Fig. S1 for a different PTC region. The same y-axis scale was used for all chromatin tracks of the same kind across all Appendix Figures.

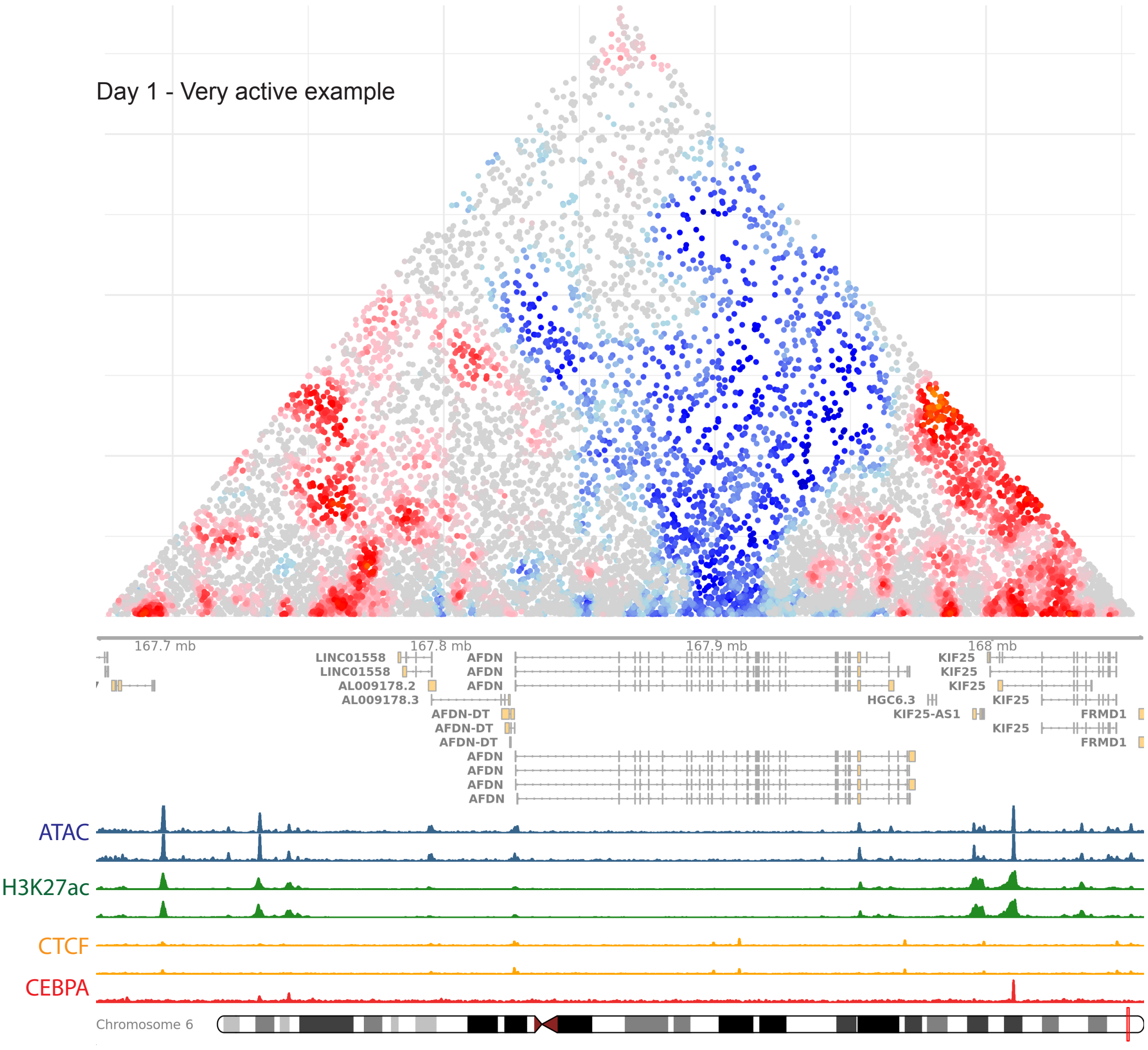

Appendix Figure S3. A Day 1 "Very active" example region in BLAER cells. Identification of "Very active" regions via SEGCOND is described in Fig. 1E & the "Materials and Methods" section. The displayed information is the same as in Appendix Fig. S1.

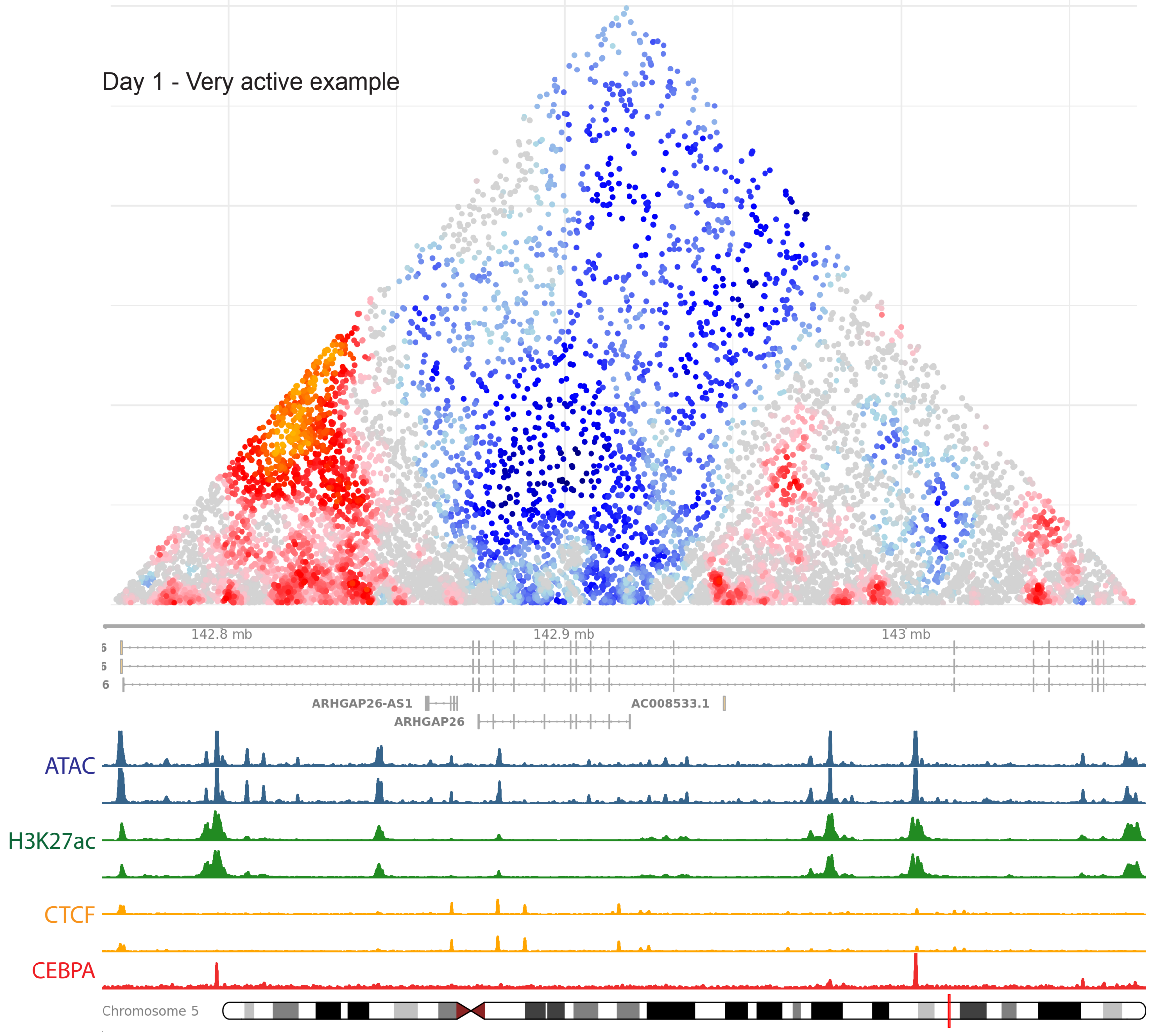

Appendix Figure S4. A Day 1 “Very active” example region in BLAER cells. Same as in Appendix Fig. S3 for a different “Very active” region.

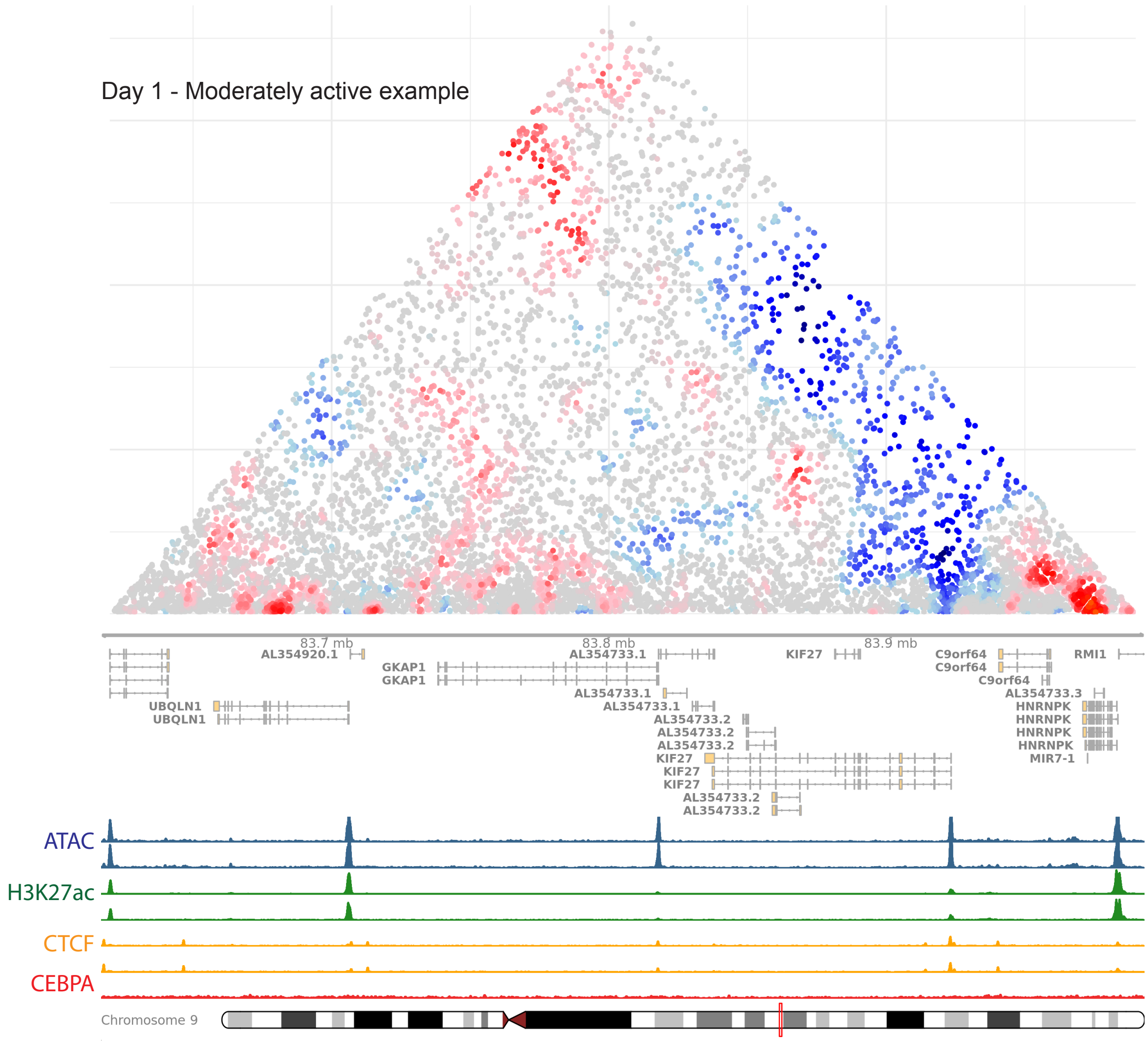

Appendix Figure S5. A Day 1 "Moderately active" example region in BLAER cells. Identification of "Moderately active" regions via SEGCOND is described in Fig. 1E & the "Materials and Methods" section. The displayed information is the same as in Appendix Fig. S1.

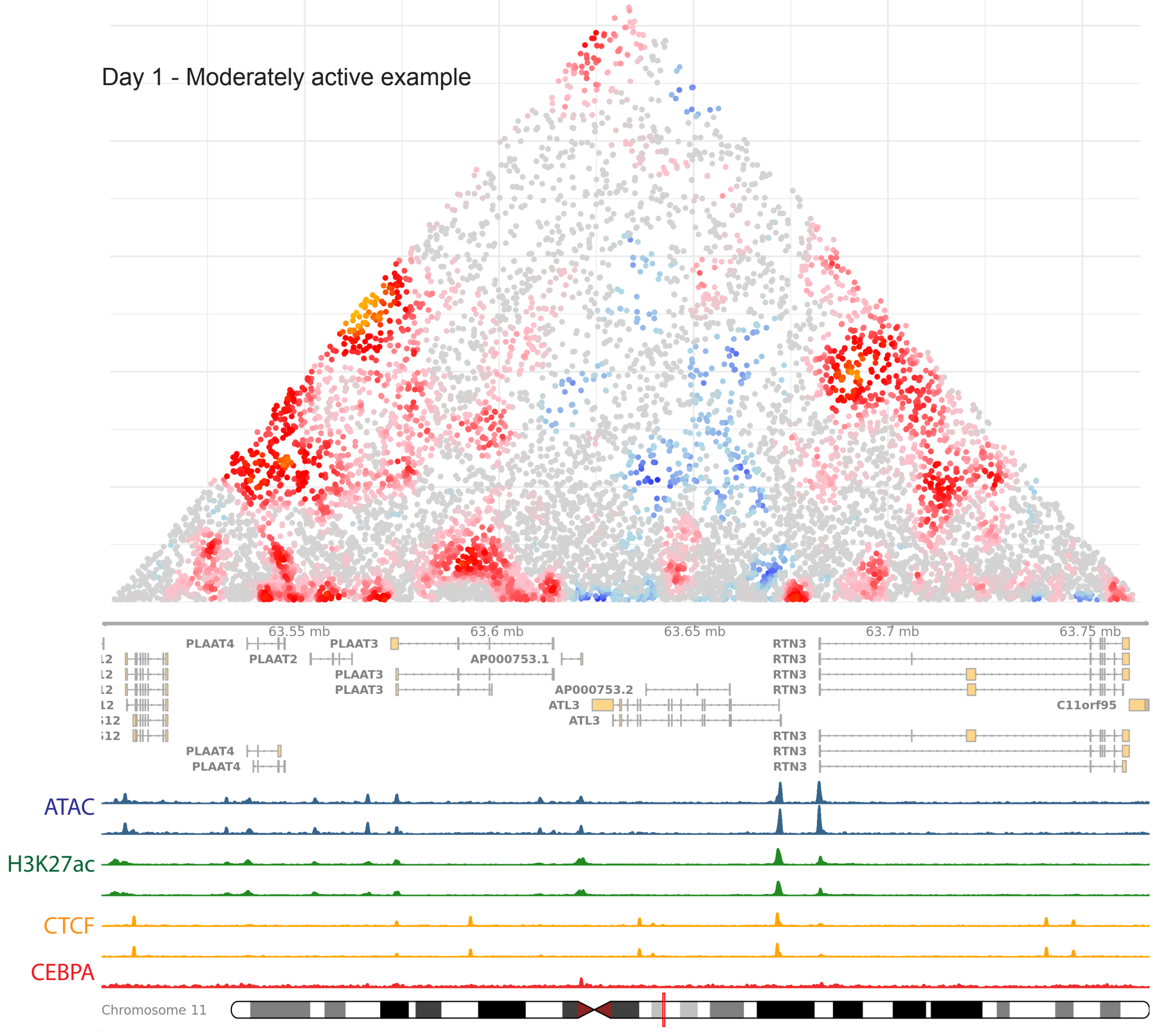

Appendix Figure S6. A Day 1 “Moderately active” example region in BLAER cells. Same as in Appendix Fig. S5 for a different “Moderately active” region.

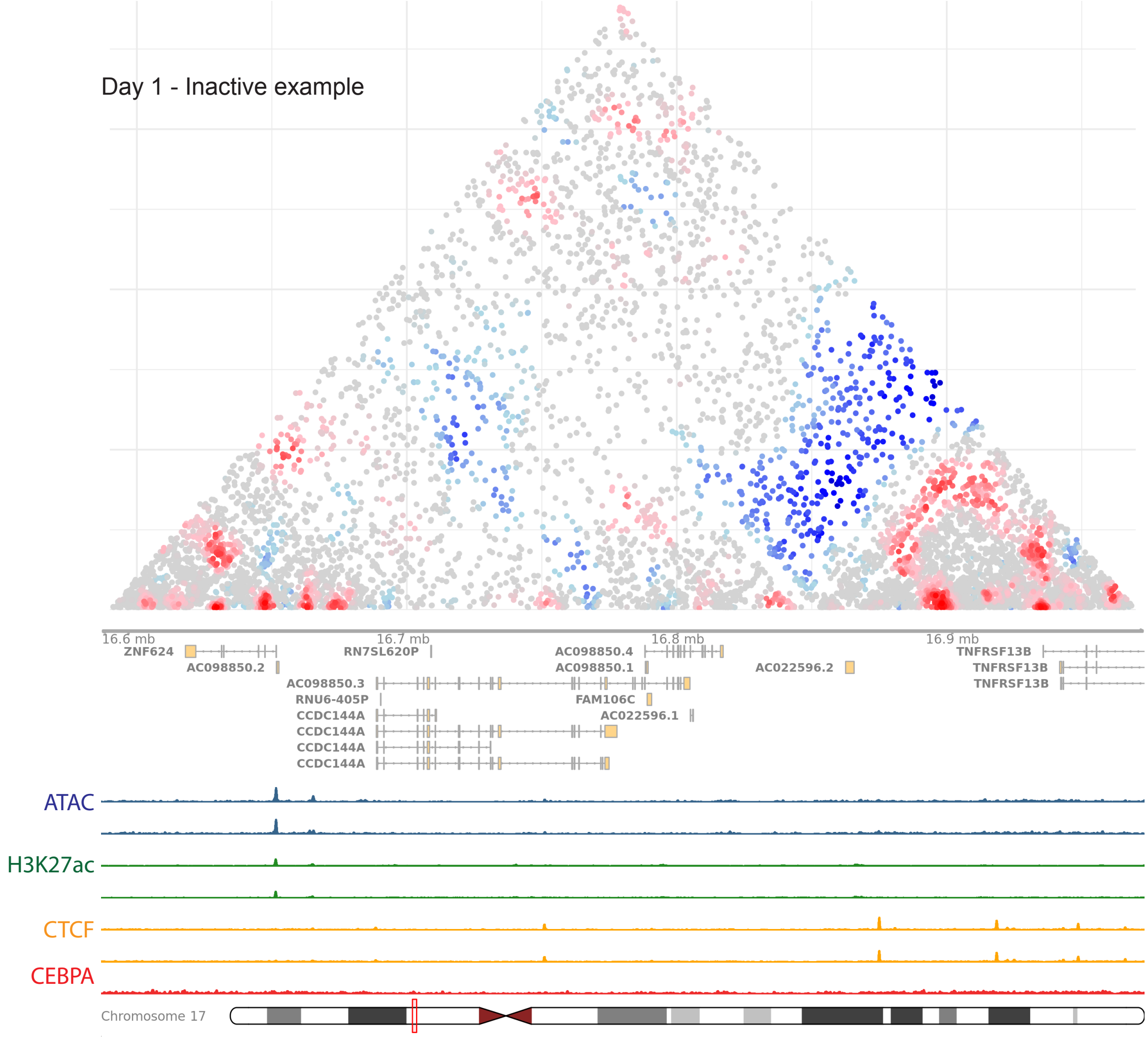

Appendix Figure S7. A Day 1 "Inactive" example region in BLAER cells. Identification of "Inactive" regions via SEGCOND is described in Fig. 1E & the "Materials and Methods" section. The displayed information is the same as in Appendix Fig. S1.

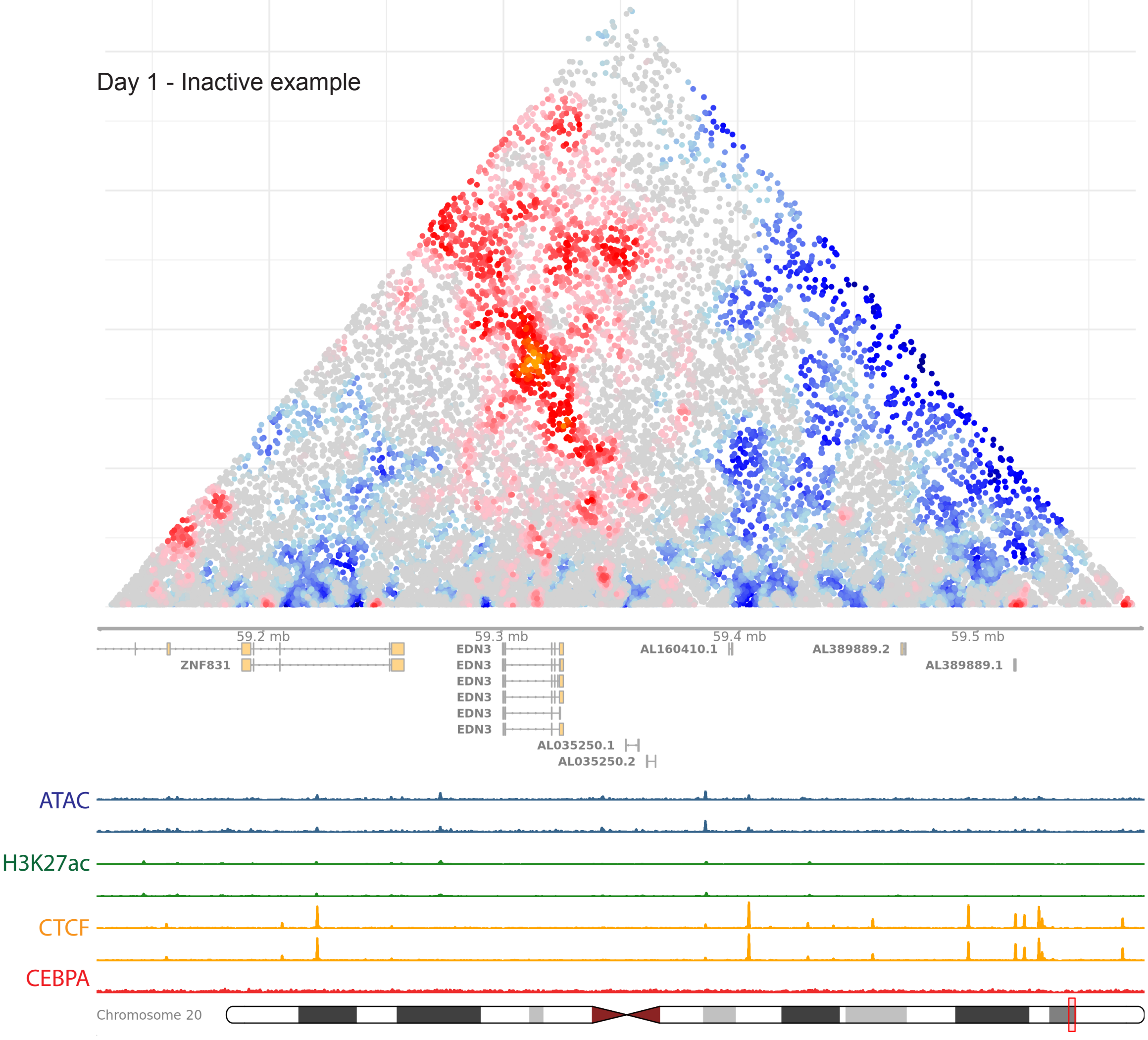

Appendix Figure S8. A Day 1 “Inactive” example region in BLAER cells. Same as in Appendix Fig. S7 for a different “Inactive” region.
