# Supplementary material for: Synergistic and antagonistic activities of IRF8 and FOS enhancer pairs during an immune-cell fate switch
Source: EMBO J. 2025 Feb 19;44(7):2025–55. doi: 10.1038/s44318-025-00380-w (PMC11961672; doi:10.1038/s44318-025-00380-w)
Supplement: Supplementary file 8 — Expanded View Figures [file 44318_2025_380_MOESM8_ESM.pdf]

# Expanded View Figures

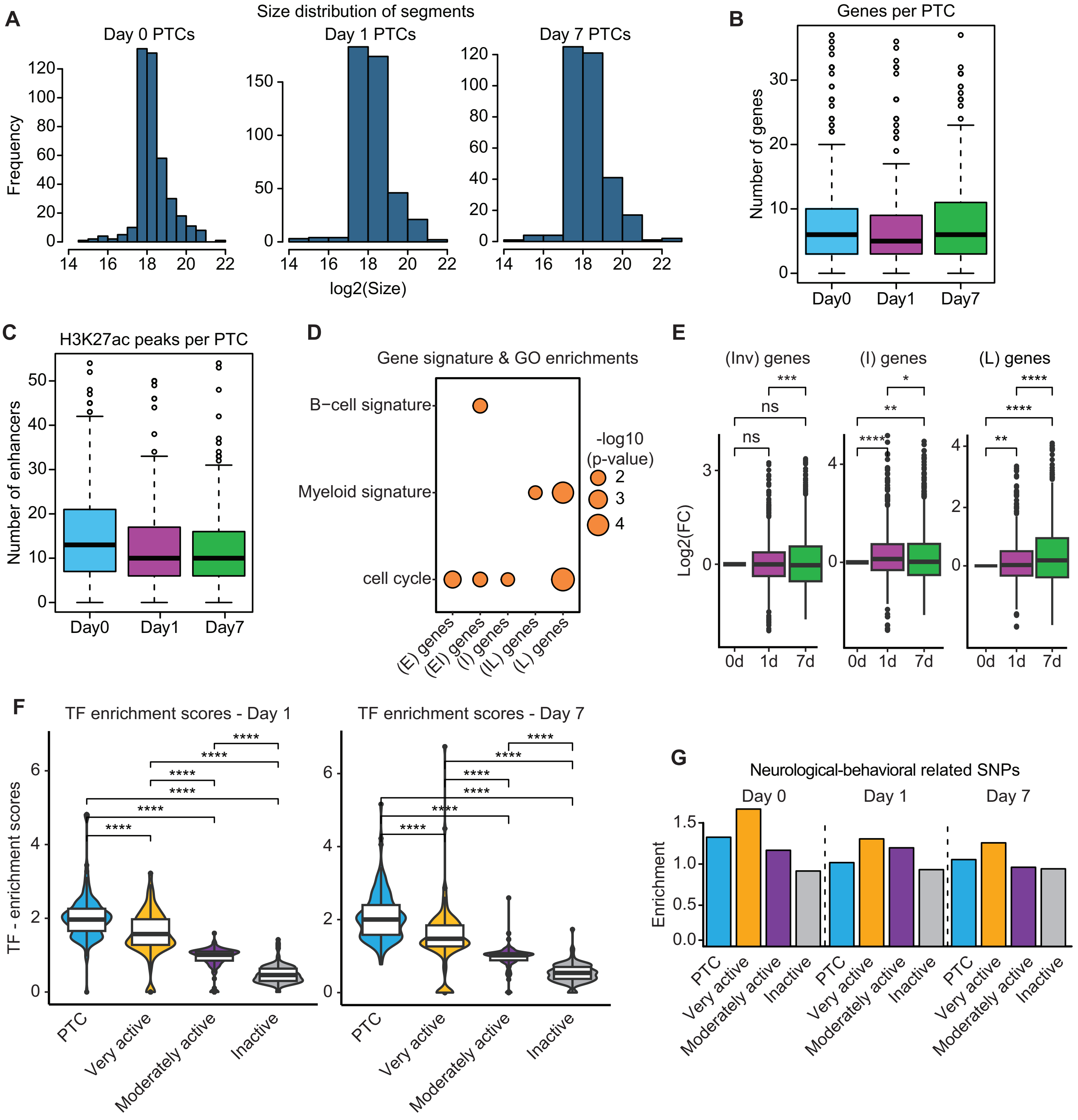

**Figure EV1. PTCs contain highly expressed, lineage-specific genes across all transdifferentiation stages.**

(**A**) Size distribution of segments within PTCs. Histograms were used to depict the size distribution of segments across the three transdifferentiation stages. $N = 415$ (Day 0), 437 (Day 1), 316 (Day 7). (**B**) Distribution of genes per PTC. Genes were filtered as in Fig. 1B. Genes found within different segments of the same PTC were pooled together. $N = 333$ (Day 0), 373 (Day 1), and 271 (Day 7). (**C**) Distribution of H3K27ac peaks per PTC. H3K27ac peaks of different segments that belong to the same PTC were pooled together. $N = 333$ (Day 0), 373 (Day 1), and 271 (Day 7). (**D**) Cell signature enrichments of genes within PTC clusters. A custom list of B-cell-specific and myeloid-specific genes was downloaded from (Monaco et al, 2019). Overlap significance between the generated gene lists and the genes within each PTC cluster was assessed via a hypergeometric distribution test. Gene ontology (GO) enrichment analysis for the genes of each PTC group was performed using gprofiler2 (Kolberg et al, 2020) with the "cell cycle" enriched GO term being depicted. The *p* value of the "cell cycle" GO term was calculated by gprofiler2. *P* values are represented as circles, with the circle area being proportional to the *P* value. (**E**) Expression dynamics of genes falling in the "Invariant", "Intermediate" and "Late" PTC clusters. Values were processed as in Fig. 1D. The paired Wilcoxon signed-rank test was used to determine statistically significant differences (ns $P$ value > 0.05; *$P$ value ≤0.05; **$P$ value ≤0.01; ***$P$ value ≤0.001; ****$P$ value ≤0.0001). $N = 1446$ (Inv genes), 573 (I genes), 680 (L genes). (**F**) Transcription factor–target enrichment scores for the four SEGCOND genomic groups at Days 1 and 7 of transdifferentiation. Scores were calculated as in Fig. 1F. Statistically significant differences were determined via a paired Wilcoxon rank-sum test (****$P$ value ≤ 0.0001). $N = 131$ for all genomic region types and timepoints. (**G**) Overlap enrichment of SEGCOND genomic categories with neurological-associated single nucleotide polymorphisms (SNPs) as in Fig. 1H. No significant overlap was observed with this SNP category. Source data are available online for this figure.

 © The Author(s)

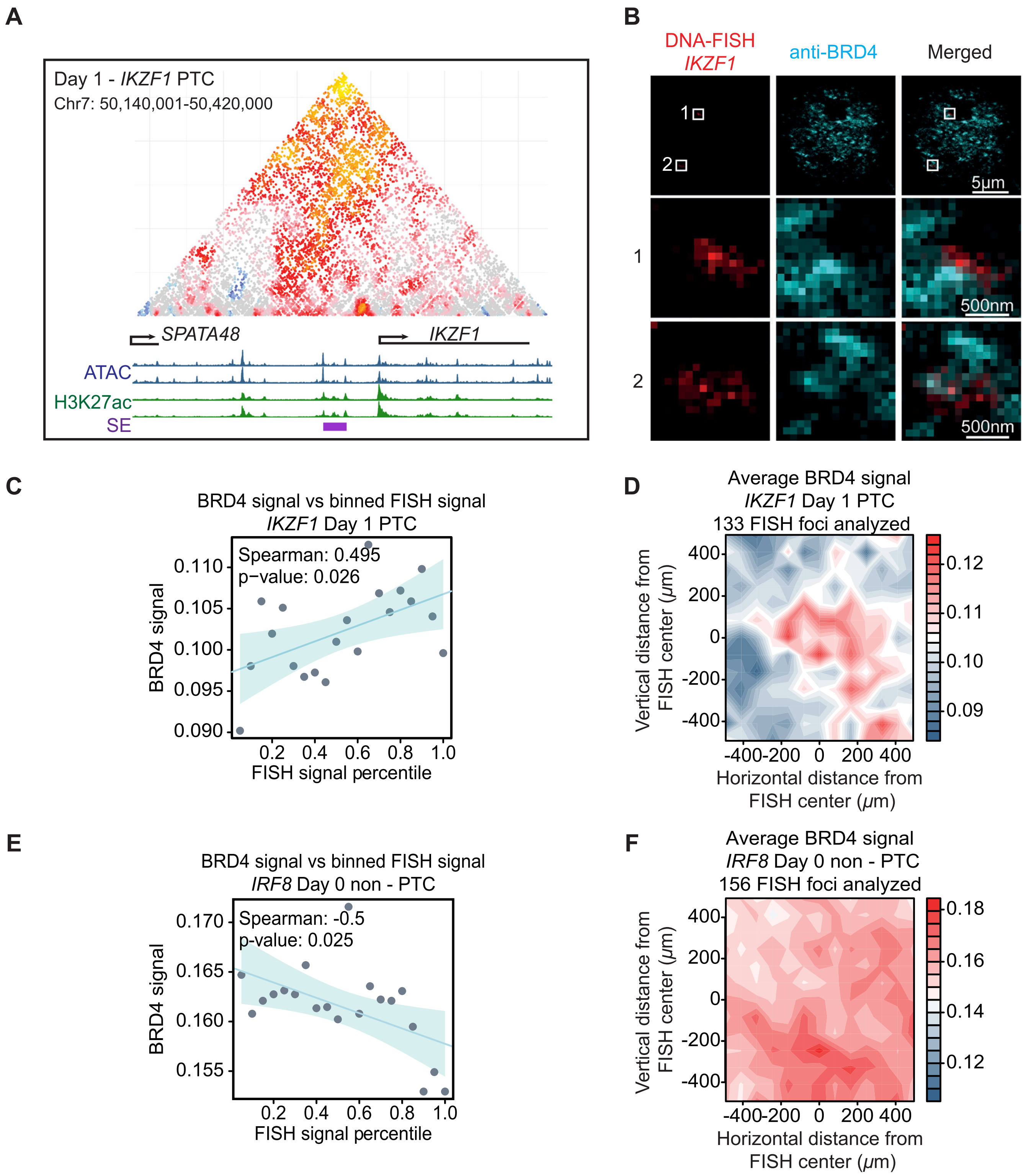


**Figure EV2. BRD4 hubs associate with active PTCs.**

(**A**) Overview of the *IKZF1* PTC at Day 1 cells. As in Fig. 2A. (**B**) DNA-FISH coupled with BRD4 immunofluorescence, targeting the *IKZF1* PTC at Day 1 cells. The third and second rows of images correspond to the zoomed regions falling within the first and second highlighted areas (white frames). (**C**) DNA-FISH and BRD4 signal correlation analysis for Day-1 *IKZF1* PTC data. Same as in Fig. 2C. (**D**) Contour plots of BRD4 signal enrichment over Day 1 *IKZF1* DNA-FISH centers. Same as in Fig. 2D. (**E**) DNA-FISH and BRD4 signal correlation analysis for Day 0 *IRF8* PTC data. Same as in Fig. 2C. Higher DNA-FISH values anti-correlate with high BRD4 values, indicating that BRD4 does not associate with the *IRF8* PTC at Day 0 cells. (**F**) Contour plots of BRD4 signal enrichment over *IRF8* Day 0 DNA-FISH centers. Same as in Fig. 2D. No enrichment of BRD4 signal at DNA-FISH centers, marking the *IRF8* PTC, can be observed at Day 0 cells.

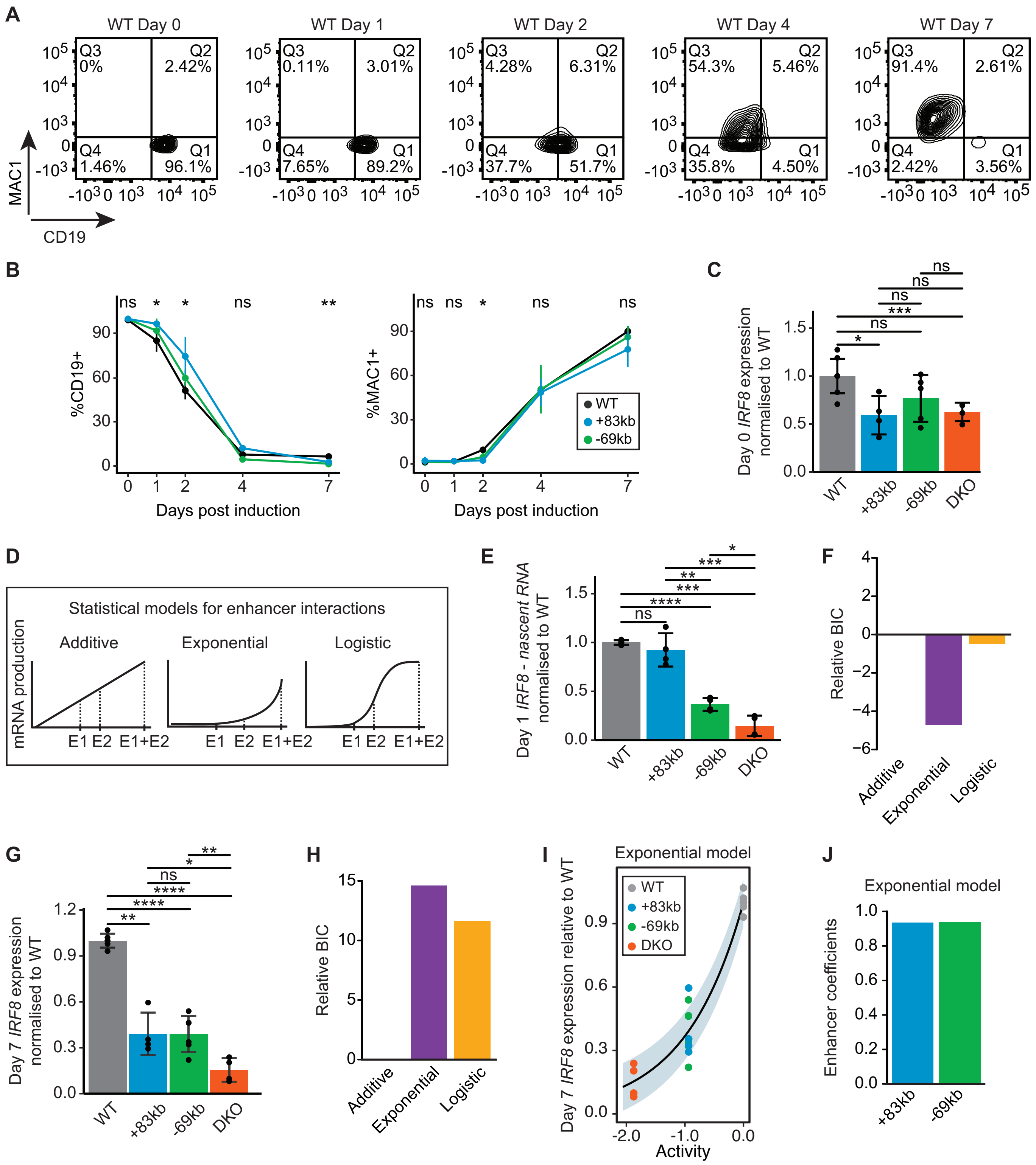
A
WT Day 0
WT Day 1
WT Day 2
WT Day 4
WT Day 7
Q3 0%
Q2 2.42%
Q4 1.46%
Q1 96.1%
Q3 0.11%
Q2 3.01%
Q4 7.65%
Q1 89.2%
Q3 4.28%
Q2 6.31%
Q4 37.7%
Q1 51.7%
Q3 54.3%
Q2 5.46%
Q4 35.8%
Q1 4.50%
Q3 91.4%
Q2 2.61%
Q4 2.42%
Q1 3.56%
MAC1
CD19
B
%CD19+
%MAC1+
Days post induction
WT
+83kb
-69kb
C
Day 0 IRF8 expression normalised to WT
WT
+83kb
-69kb
DKO
D
Statistical models for enhancer interactions
mRNA production
Additive
Exponential
Logistic
E1 E2 E1+E2
E
Day 1 IRF8 - nascent RNA normalised to WT
F
Relative BIC
Additive
Exponential
Logistic
G
Day 7 IRF8 expression normalised to WT
H
Relative BIC
I
Exponential model
Day 7 IRF8 expression relative to WT
Activity
DKO
J
Exponential model
Enhancer coefficients

 © The Author(s)

**Figure EV3. The -69kb and + 83 kb *IRF8* enhancers synergize to drive high *IRF8* expression levels.**

(**A**) FACS gating strategy used to determine CD19+ and MAC1+ cells during transdifferentiation. Cells falling within Q1 & Q2 were deemed as CD19 + , whereas cells within Q3 & Q4 as MAC1 + . (**B**) Transdifferentiation kinetics of *IRF8* -69kb KO and +83KO cells compared to BLAER cells, monitored via FACS. The mean ± s.d. is depicted for each timepoint. Statistical significance was determined per timepoint using a two-way ANOVA test, including the transdifferentiation batch as a covariate (ns *P* value > 0.05; **P* value ≤ 0.05; ***P* value ≤0.01). *N* = 3 (WT, −69 kb, +83 kb Days 0, 1, 2, and 7), and 2 (WT, −69 kb, +83 kb, Day 4) biological replicates. (**C**) Day 0 *IRF8* expression levels in WT and KO cells. *IRF8* expression before CEBPA induction was quantified via RT-qPCR and normalized against *GUSB* expression. To account for batch-to-batch variations, the expression values from each batch were normalized to the mean WT expression values within the same batch. The mean ± s.d. is depicted, with individual measurements included as points. Statistical significance was determined using a Student's *t* test (ns *P* value > 0.05; **P* value ≤0.05; ****P* value ≤0.001). *N* = 8 (WT), 5 (−69 kb), and 4 ( + 83 kb, DKO) biological replicates. (**D**) Overview of statistical models adapted from (Dukler et al, 2017). In brief, the additive model assumes that each enhancer independently adds its activity, linearly increasing gene expression levels. The exponential model assumes synergy between enhancers, leading to exponential increases in gene expression levels. Finally. the logistic model predicts that transcription occurs in a low-energy state, with each enhancer independently reducing the energy threshold required to reach it. (**E**) Quantification of newly synthesized *IRF8* mRNA after CEBPA induction. Simultaneous to CEBPA activation, cells were fed an ethylene uridine (EU) ribonucleotide homolog (Click-iT™ Nascent RNA Capture Kit, #C10365), which is incorporated into newly synthesized mRNA molecules and allows their isolation. Isolated nascent RNA was used in RT-qPCR experiments to determine *IRF8* nascent RNA levels in WT and KO cells. Nascent RNA levels of *IRF8* were normalized as in Fig. 3E. The mean ± s.d. is depicted, alongside individual measurements as points. Statistical significance was determined using a Student's *t* test (ns *P* value > 0.05; **P* value ≤0.05; ***P* value ≤0.01; ****P* value ≤0.001; *****P* value ≤0.0001). *N* = 4 biological replicates for all samples. (**F**) Evaluation of best statistical model fit for Day 1 *IRF8* nascent RNA data. As in Fig. 3F. Both the additive and logistic models were found to be good fits. (**G**) Day 7 *IRF8* expression levels of all generated KO lines and WT cells. Same as Fig. 3E. Statistical significance was determined using a Student's *t* test (ns *P* value > 0.05; **P* value <= 0.05; ***P* value ≤0.01; *****P* value ≤0.0001). *N* = 6 (WT, −69 kb), and 4 ( + 83 kb, DKO) biological replicates. (**H**) Evaluation of best statistical model fit for Day 7 *IRF8* expression data. As in Fig. 3F, except for the exponential model being the best fit for the dataset. (**I**) Exponential model fit to *IRF8* Day 7 expression data. Same as in Fig. 3G. (**J**) Exponential model enhancer coefficients for *IRF8* Day 7 expression data. As in Fig. 3H, except that the model assigns very similar activities to the two enhancers. Source data are available online for this figure.

© The Author(s)

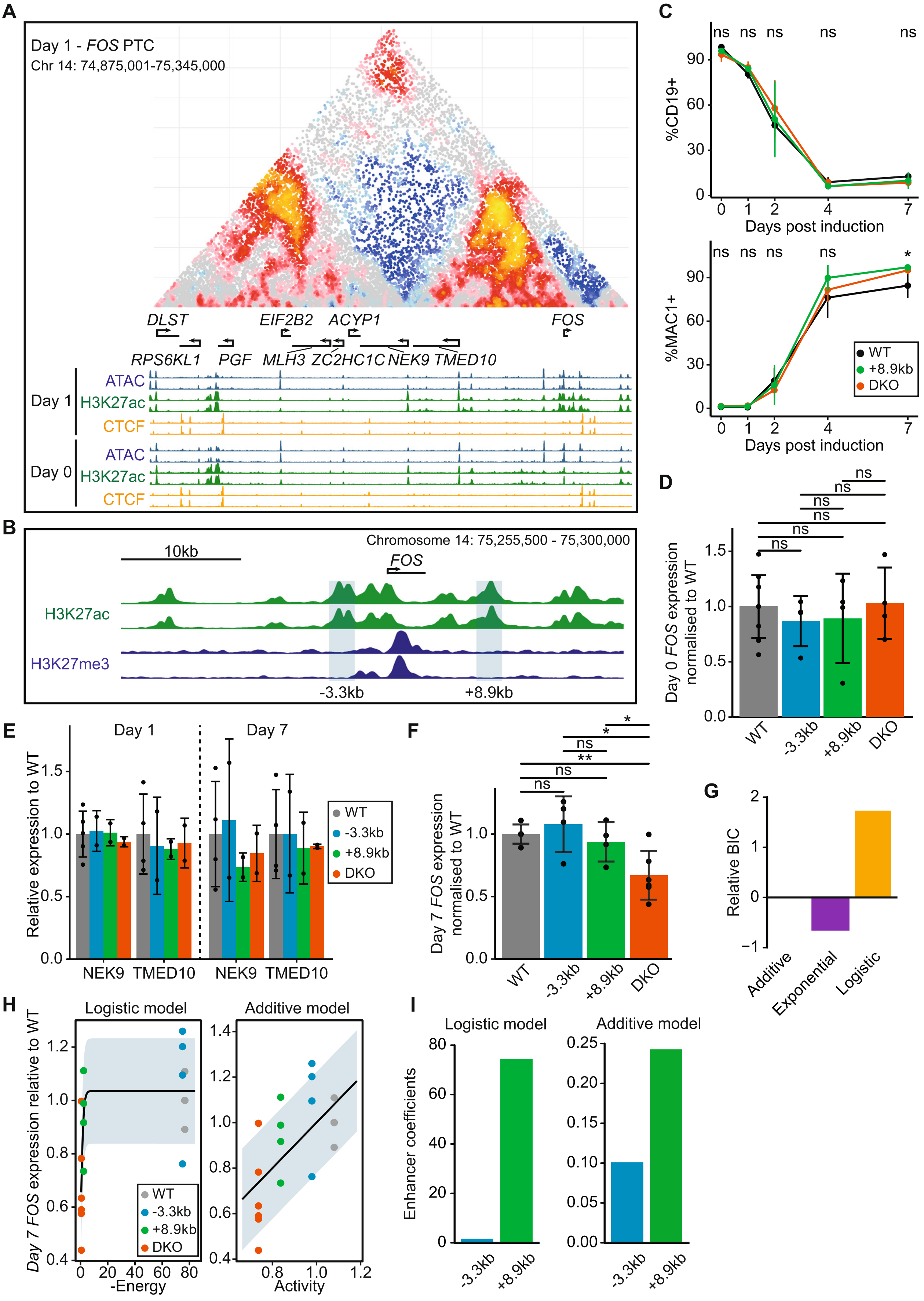
A
Day 1 - FOS PTC
Chr 14: 74,875,001-75,345,000
DLST
EIF2B2
ACYP1
FOS
RPS6KL1
PGF
MLH3
ZC2HC1C
NEK9
TMED10
Day 1
ATAC
H3K27ac
CTCF
Day 0
ATAC
H3K27ac
CTCF
B
Chromosome 14: 75,255,500 - 75,300,000
10kb
FOS
H3K27ac
H3K27me3
-3.3kb
+8.9kb
C
%CD19+
Days post induction
%MAC1+
Days post induction
WT
+8.9kb
DKO
D
Day 0 FOS expression normalised to WT
WT
-3.3kb
+8.9kb
DKO
E
Day 1
Day 7
Relative expression to WT
NEK9
TMED10
WT
-3.3kb
+8.9kb
DKO
F
Day 7 FOS expression normalised to WT
G
Relative BIC
Additive
Exponential
Logistic
H
Logistic model
Additive model
Day 7 FOS expression relative to WT
-Energy
Activity
I
Logistic model
Additive model
Enhancer coefficients

 © The Author(s)

**Figure EV4. The -3.3 kb and + 8.9 kb *FOS* enhancers cooperate at Day 7 cells, safeguarding *FOS* expression.**

(**A**) Overview of the *FOS* PTC on Day 1 of transdifferentiation. As in Fig. 2A. CTCF ChIP-seq tracks are also displayed. (**B**) Overview of the H3K27ac and H3K27me3 marks in the *FOS* PTC at Day 1 cells. Two H3K27ac and two H3K27me3 ChIP-seq tracks of two biological replicates are depicted. The −3.3 kb enhancer isn't decorated with the repressive histone mark H3K27me3. (**C**) Transdifferentiation kinetics of *FOS* + 8.9 kb KO and DKO cells compared to BLAER cells, monitored via FACS. The mean ± s.d. of CD19+ and MAC1+ cells is shown. Statistical significance per timepoint was determined using a two-way ANOVA test, factoring in transdifferentiation batches (ns *P* value > 0.05; **P* value ≤0.05). $N = 4$ (WT Days 0, 1, 2, DKO all timepoints), 3 (WT Days 4, 7, +8.9 kb Days 0, 1, 2), and 2 ( + 8.9 kb Days 4, 7) biological replicates. (**D**) Day 0 *FOS* expression levels in WT and KO cells. Normalization was performed as in Fig. 4G. Statistical significance was determined using a Student's *t* test (ns *P* value > 0.05). $N = 9$ (WT), and 4 (−3.3 kb, +8.9 kb, DKO) biological replicates. (**E**) Day 1 and Day 7 expression levels of other genes within the *FOS* PTC. *NEK9* and *TMED10* expression levels were quantified via RT-qPCR and normalized against GUSB expression. The normalized expression values were then divided by the average expression of WT cells. The mean ± s.d. are depicted for each gene, sample, and timepoint. Individual measurements are also depicted as points. Statistical significance of expression differences between cell lines was determined using the Student's *t* test, and no significant hits were found. $N = 5$ (WT, NEK9 Day 1), 4 (WT, NEK9 Day 7, TMED10 Days 1 and 7), and 2 (all other samples) biological replicates. (**F**) Day 7 *FOS* expression levels of KO and WT cells. As in Fig. 4G. Statistical significance was determined using a Student's *t* test (ns *P* value > 0.05; **P* value ≤0.05; ***P* value ≤0.01) $N = 6$ (DKO), 5 (WT), and 4 (−3.3 kb & +8.9 kb) biological replicates. (**G**) Evaluation of best model fit for Day 7 *FOS* expression data. As in Fig. 3F. Both the logistic and additive models were deemed valid. (**H**) Logistic and additive model fits to *FOS* Day 7 expression data. Same as in Fig. 3G. (**I**) Logistic and additive model enhancer coefficients for Day 7 *FOS* expression data. The +8.9 kb enhancer activity is proposed to be higher than the activity of the −3.3 kb enhancer. Source data are available online for this figure.

© The Author(s)

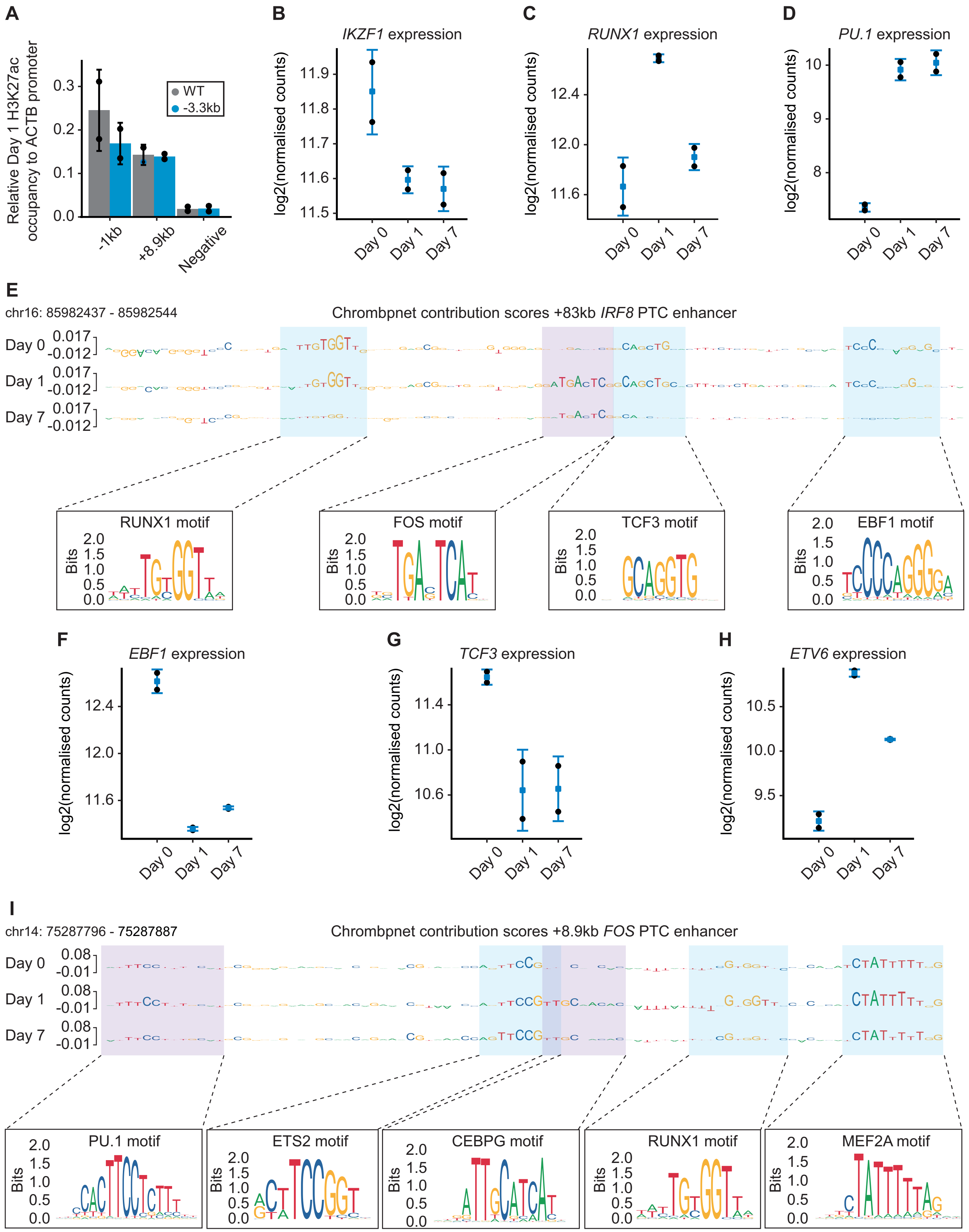
A
Relative Day 1 H3K27ac occupancy to ACTB promoter
WT
-3.3kb
-1kb
+8.9kb
Negative
B
IKZF1 expression
log2(normalised counts)
Day 0
Day 1
Day 7
C
RUNX1 expression
D
PU.1 expression
E
chr16: 85982437 - 85982544
Chrombpnet contribution scores +83kb IRF8 PTC enhancer
RUNX1 motif
FOS motif
TCF3 motif
EBF1 motif
Bits
F
EBF1 expression
G
TCF3 expression
H
ETV6 expression
I
chr14: 75287796 - 75287887
Chrombpnet contribution scores +8.9kb FOS PTC enhancer
PU.1 motif
ETS2 motif
CEBPG motif
RUNX1 motif
MEF2A motif

 © The Author(s)

**Figure EV5. In silico analysis of *IRF8* and *FOS* PTC enhancer properties.**

(**A**) H3K27ac ChIP-qPCR of *FOS* PTC enhancers at Day 1 of transdifferentiation in WT and *FOS* -3.3 kb cells. Primers targeting the +8.9 kb enhancer and a region 1 kb upstream of the *FOS* transcription start site were used. Data was analyzed as in Fig. 5A. $N = 2$ biological replicates for each region and cellular background. (**B**) Expression profile of *IKZF1* across transdifferentiation. Log2 transformed, DESeq2 variance stabilized counts of two independent RNA-seq replicates are depicted. The mean ± s.d. is depicted in blue, while individual measurements are labeled as points in black. $N = 2$ biological replicates for each timepoint. (**C**) Expression profile of *RUNX1* across transdifferentiation. As in Fig. EV5B. (**D**) Expression profile of *PU.1* across transdifferentiation. As in Fig. EV5B. (**E**) ChromBPnet contribution scores in the +83 kb *IRF8* PTC enhancer at all transdifferentiation stages. As in Fig. 5E. (**F**) Expression profile of *EBF1* across transdifferentiation. As in Fig. EV5B. (**G**) Expression profile of *TCF3* across transdifferentiation. As in Fig. EV5B. (**H**) Expression profile of *ETV6* across transdifferentiation. As in Fig. EV5B. (**I**) ChromBPnet contribution scores in the +8.9 kb *FOS* PTC enhancer at all transdifferentiation stages. As in Fig. 5E. Source data are available online for this figure.
